# Deep sequencing of the microRNA transcriptome in current, former, and never smokers with lung adenocarcinoma

**DOI:** 10.1186/1753-6561-6-S6-P38

**Published:** 2012-10-01

**Authors:** Teresa W Wang, Josh D Campbell, Lingqi Luo, Gang Liu, Ji Xiao, Marc E Lenburg, Steven A Belinsky, Avrum Spira

**Affiliations:** 1 Bioinformatics Program, Boston University, Boston, MA 02215, USA; 2 Section of Computational Biomedicine, Boston University School of Medicine, Boston, MA 02118, USA; 3 Department of Pathology and Laboratory Medicine, Boston University School of Medicine, Boston, MA 02118, USA; 4 Lung Cancer Program, Lovelace Respiratory Research Institute, Albuguergue, NM 87108, USA; 5 Section of Pulmonary, Critical Care and Allergy, Boston University School of Medicine, Boston, MA 02118, USA

## Background

Lung cancer is the leading cause of cancer-related death in the USA. While smoking is a major risk factor, about 10% to 15% of cases arise in never smokers. Small non-coding RNAs such as microRNAs (miRNAs) often act as oncogenes or tumor suppressors to regulate gene expression during disease, and represent attractive targets for lung cancer risk assessment and therapeutic intervention. We therefore sought to characterize the tumor-associated miRNA transcriptome of current, former and never smokers.

## Materials and methods

Total RNA was isolated from the tumor and adjacent-normal pairs (tumor purity ≥70%; normal tissue is collected most distant from the tumor at time of resection) of current (*n* = 8), former (*n* = 11) and never (*n* = 14) smokers with adenocarcinoma. Small RNA libraries were generated and then multiplexed 7 to 8 per lane for sequencing on the Illumina HiSeq 2000. Through a custom miRNA sequencing analysis pipeline, reads were trimmed, size-selected, and mapped to an assembly of the human genome reference (hg 19) using Bowtie. Counts per mature miRNA from aligned reads were computed using Bedtools and a list of genomic features retrieved from miRBase (version 17). Finally, differential expression analysis was conducted using Student's *t* test and ANOVA.

## Results

Small RNA sequencing generated an average of 10 million high-quality miRNA reads per sample. Among the 1,906 mature miRNAs examined, 554 miRNAs had at least an average of 20 counts across all 44 samples. 94 miRNAs (false discovery rate *q*<0.25) are differentially expressed in tumors of ever smokers (*n* = 19) compared with never smokers (*n* = 14). This includes miRNA-320b, which appears to be highly activated in current and former smokers, but unchanged in never smokers. Several miRNAs, including miRNA-21 and miRNA-182, were significantly upregulated in tumors compared with the adjacent-normal, irrespective of smoking status.

## Conclusions

Using small RNA sequencing, we are comprehensively profiling the miRNA transcriptome in current, former and never smoker lung adenocarcinoma. We have demonstrated that subsets of miRNAs are markedly dysregulated in tumors when compared to their adjacent-normal counterparts. Some of these profiles may be unique to lung cancers cases that arise in current and former smokers. These results will supplement our analysis of large RNA libraries prepared from the same set of tissues to extend our knowledge of the lung cancer transcriptome, to identify key microRNA-mRNA interactions underlying lung carcinogenesis, and to better understand general mechanisms of lung carcinogenesis as well as those that are specific to carcinogenesis in the presence or absence of tobacco smoke exposure.

